# Differential response to right ventricular extrastimuli from the base and apex during long RP′ supraventricular tachycardia

**DOI:** 10.1002/joa3.13214

**Published:** 2025-01-07

**Authors:** Hironori Nakamura, Hidehira Fukaya, Naruya Ishizue, Jun Kishihara, Junya Ako

**Affiliations:** ^1^ Department of Cardiovascular Medicine Kitasato University School of Medicine Sagamihara Japan

**Keywords:** atrioventricular nodal reentrant tachycardia, bystander, nodoventricular pathway, paradoxical reset

## Abstract

We report a case of long RP′ tachycardia diagnosed as fast–slow atrioventricular nodal reentrant tachycardia (AVNRT) with a bystander nodoventricular pathway (NVP). Differential responses to right ventricular extrastimuli from the base and apex highlighted the anatomical proximity of the NVP attachment, contributing to the diagnosis.

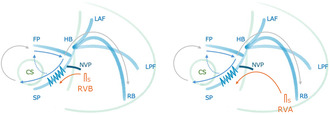

A 16‐year‐old male presented at our hospital due to a history of syncope. Pre‐procedural evaluations revealed no evidence of structural heart disease. An electrophysiological study (EPS) under local anesthesia showed stable hemodynamics. The baseline AH and HV intervals were measured at 93 and 40 ms, respectively. A long RP′ tachycardia with a cycle length of 440 m spontaneously occurred during the EPS. The proximal coronary sinus was identified as the earliest atrial activation site during the tachycardia. An extrastimulus was applied from the apex of the right ventricle (RVA) during the refractory period of the His bundle (HB), resulting in the resetting of the tachycardia (Figure 1A). The application of overdrive pacing from the base of the right ventricle (RVB) resulted in the cessation of tachycardia without atrial capture, with a total pacing prematurity (TPP) of 137 ms (Figure 1B). Furthermore, an extrastimulus delivered from the RVB slightly after the HB refractory period also resulted in the resetting of the tachycardia (Figure 2A). However, an extrastimulus delivered slightly in advance of the HB refractory period from the same site resulted in the cessation of the tachycardia reset phenomenon (Figure 2B). This phenomenon was not reproducible by an extrastimulus from the RVA.

**FIGURE 1:**
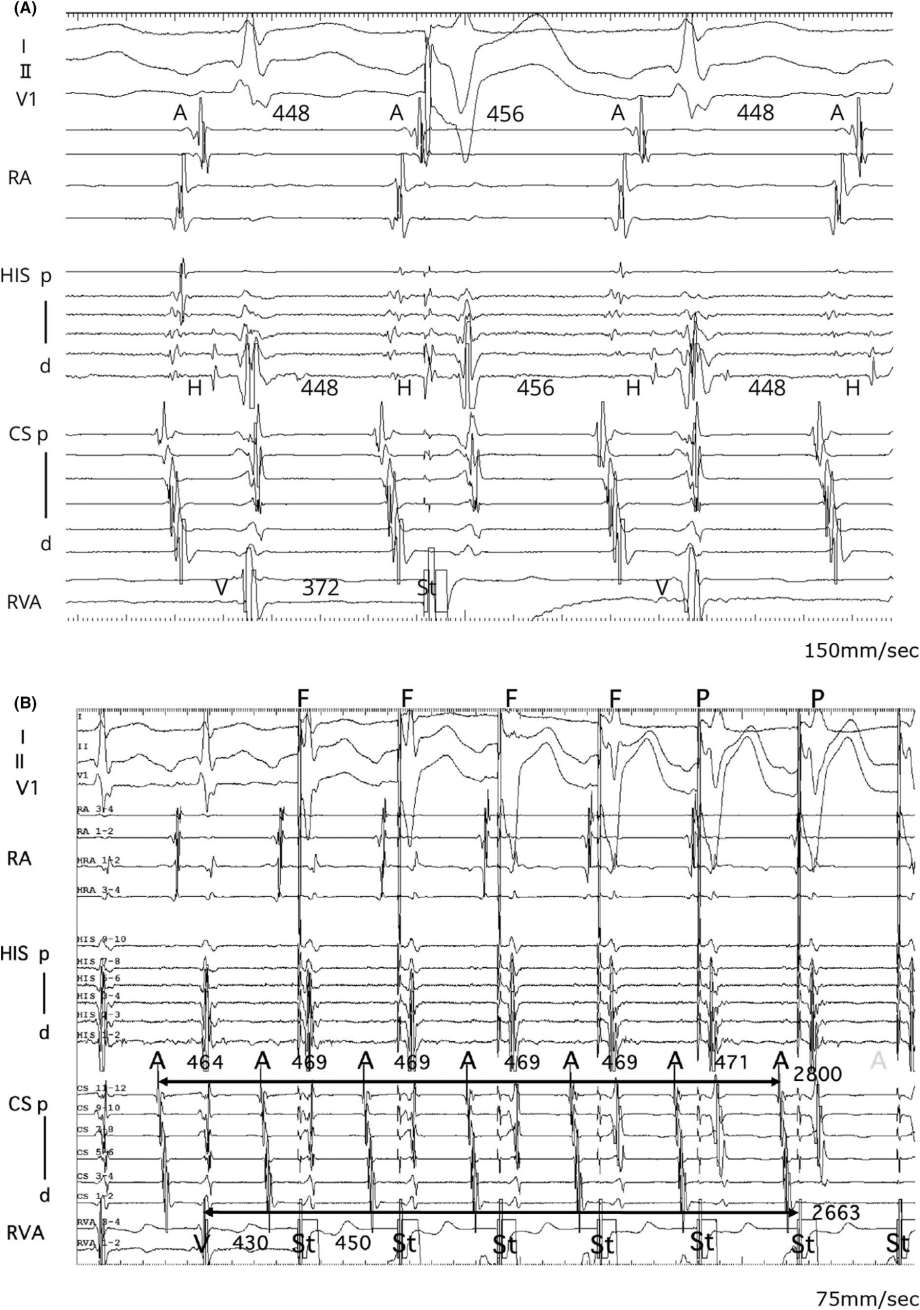
(A) A ventricular extrastimulus delivered from RVA during the HB refractory period. (B) Ventricular overdrive pacing from RVA. A, atrial potential; CS, coronary sinus; d, distal; F, fusion QRS; H, His potential; HIS, a bundle of His; p, proximal; P, paced QRS; RA, right atrium; RVA, apex of right ventricle; RVB, base of right ventricular; St, programmed stimulation.

**FIGURE 2:**
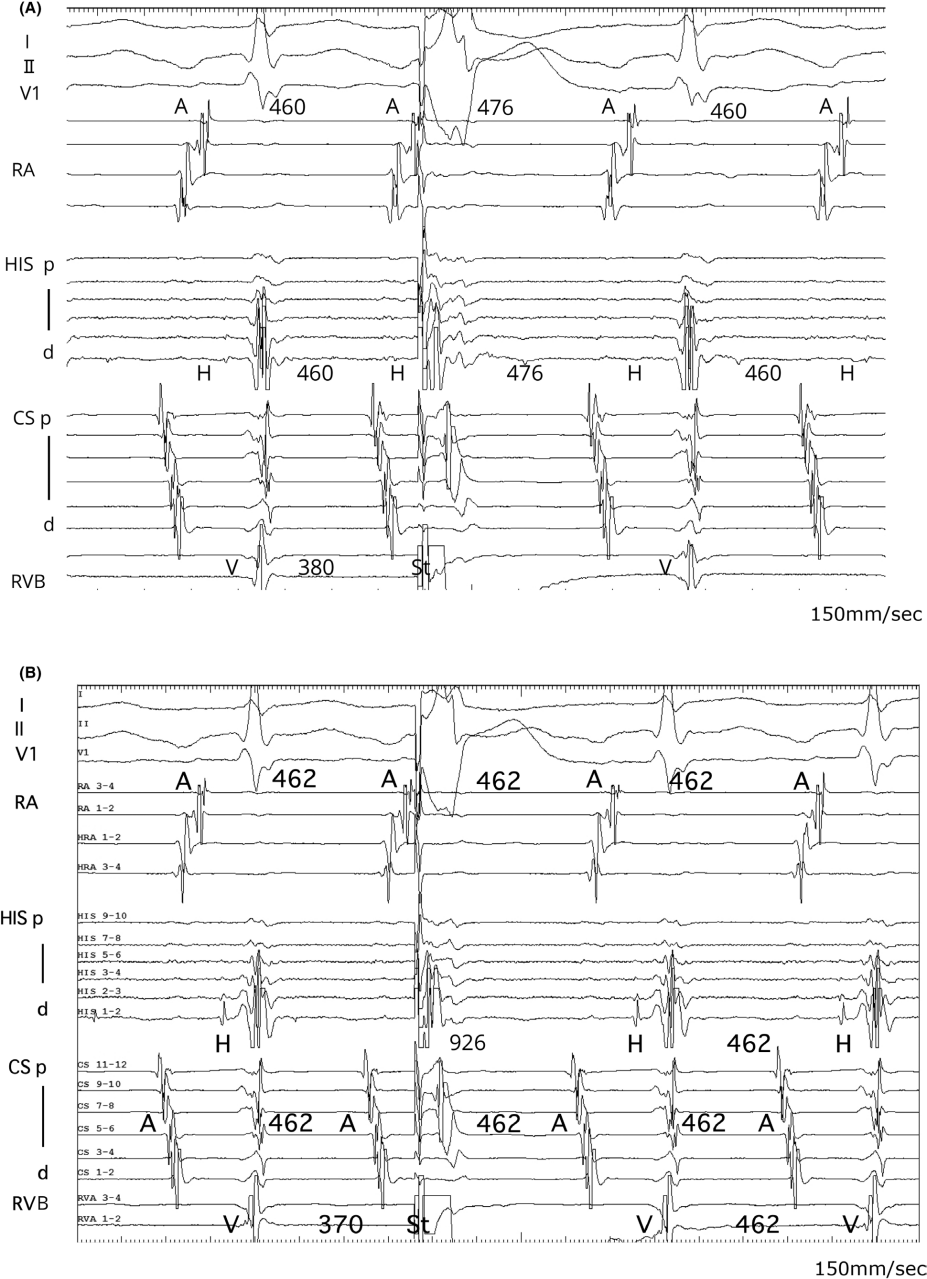
(A) A ventricular extrastimulus delivered from the RVB during the HB refractory period also reset the tachycardia, resulting in a prolongation of both the A–A interval and the H–H interval. (B) An extrastimulus timed slightly before the HB refractory period from the same site resulted in the disappearance of the tachycardia resetting phenomenon. A, atrial potential; CS, coronary sinus; d, distal; F, fusion QRS; H, His potential; HIS, a bundle of His; p, proximal; P, paced QRS; RA, right atrium; RVA, apex of right ventricle; RVB, base of right ventricular; St, programmed stimulation.

Termination of SVT without atrial capture during ventricular overdrive pacing ruled out atrial tachycardia (AT).^1^ A ventricular extrastimulus, introduced within the HB refractory period during tachycardia, reset both the atrial excitation and the tachycardia itself. This finding suggests the presence of an accessory pathway (AP). However, progressively shortening the coupling interval of an extrastimulus from the RVB resulted in the disappearance of the resetting phenomenon, known as paradoxical reset, which indicates that the AP is a bystander.^2^, ^3^, ^4^ This phenomenon was not observed with extrastimulus from the RVA, suggesting that the attachment site of the nodoventricular pathway (NVP) is located closer to the RVB than RVA or other conduction systems. Furthermore, extrastimuli delivered from the RVB during the His refractory period resulted in more pronounced A–A interval changes and tachycardia resetting compared with those delivered from the RVA. This also suggests that the attachment site of the NVP might be closer to the RVB than to the RVA. Specifically, conduction from an extrastimulus at the RVB occurred within the effective refractory period of the NVP, readily leading to an AP block. In contrast, conduction from an extrastimulus at the RVA took longer to reach the NVP, making an AP block less likely. In this case, RVA overdrive pacing was not evaluated; however, comparing the TPP during entrainment from the apex and base of the RV would have been extremely valuable in assessing the location of the ventricular attachment of the NVP.

While paradoxical reset suggests the AP is a bystander, it does not definitively exclude the possibility of orthodromic reciprocating tachycardia (ORT). In cases where the AP has decremental conduction properties, it may mimic a paradoxical reset. Therefore, it is important to ensure that the paradoxical reset is a reproducible finding, which requires performing pacing from both the RVB and RVA to assess for any changes in behavior. Particularly when performing EPS under local anesthesia, minor variations in the tachycardia cycle length were observed due to factors such as pain, patient movement, and autonomic modulation induced by isoproterenol infusion, highlighting the need to thoroughly evaluate reproducibility. In this case, minor variations were similarly observed. However, significant fluctuations in the tachycardia cycle length were not confirmed. Based on the reproducibility of the paradoxical reset and other findings, the arrhythmia was identified as fast–slow AVNRT with a bystander concealed NVP. Additionally, other observations, such as atrioventricular block during tachycardia or HB dissociation during ventricular overdrive pacing, should be considered to comprehensively rule out ORT.

Highly specific and reproducible findings are essential for achieving a definitive diagnosis and determining the most appropriate treatment strategy. Recognizing the presence and function of the AP can guide the selection of suitable ablation therapies or other interventions. Moreover, identifying the location of the NVP enables the development of more targeted treatment strategies.^5^ In this case, slow pathway ablation was successfully performed, as the arrhythmia was suspected to be AVNRT. Estimating the anatomical location of the NVP may aid in identifying the optimal ablation site in cases of NVP‐associated tachycardia. Pacing from both the apex and base of the right ventricle can assist in assessing the presence and anatomical position of accessory pathways.

## CONFLICT OF INTEREST STATEMENT

Authors declare no conflict of interests for this article.

## ETHICS APPROVAL

The research related to human use has complied with all the relevant national regulations and institutional policies and is in accordance with the tenets of the Helsinki Declaration and has been approved by the author's institutional review board or equivalent committee.

## PATIENT CONSENT STATEMENT

Informed consent has been obtained from all individuals included in this study.

## Data Availability

The datasets generated and/or analyzed during the current study are available from the corresponding author upon reasonable request.

## References

[1] Maruyama M , Uetake S , Miyauchi Y , Seino Y , Shimizu W . Analyses of the mode of termination during diagnostic ventricular pacing to differentiate the mechanisms of supraventricular tachycardias. JACC Clin Electrophysiol. 2017;3(11):1252–1261. 29759621 10.1016/j.jacep.2017.05.014

[2] Sakai S , Nagashima K , Kaneko Y , Maruyama M . A narrow QRS complex tachycardia: what is the mechanism? Heart Rhythm. 2022;19(9):1557–1558. 35595019 10.1016/j.hrthm.2022.05.018

[3] Nagashima K , Maruyama M , Kaneko Y , Sakai S , Sekihara T , Kawaji T , et al. Systematic observation‐based diagnosis of atrioventricular nodal reentrant tachycardia with a bystander concealed nodoventricular pathway. J Arrhythm. 2024;40(1):131–142. 38333409 10.1002/joa3.12976 PMC10848616

[4] Nagashima K , Michaud GF , Ho RT , Okumura Y . SVT quest: the adventure diagnosing narrow QRS tachycardia. J Arrhythm. 2024;40(4):767–785. 39139886 10.1002/joa3.13112 PMC11317754

[5] Nazer B , Walters TE , Dewland TA , Naniwadekar A , Koruth JS , Najeeb Osman M , et al. Variable presentations and ablation sites for manifest nodoventricular/nodofascicular fibers. Circ Arrhythm Electrophysiol. 2019;12(9):e007337. 31505948 10.1161/CIRCEP.119.007337

